# *Scedosporium prolificans* Septic Arthritis and Osteomyelitis of the Hip Joints in an Immunocompetent Patient: A Case Report and Literature Review

**DOI:** 10.1155/2017/3809732

**Published:** 2017-01-10

**Authors:** Luca Daniele, Michael Le, Adam Franklin Parr, Lochlin Mark Brown

**Affiliations:** Department of Orthopaedics, Gold Coast Health Service, Gold Coast University Hospital, 1 Hospital Blvd., Southport, QLD 4215, Australia

## Abstract

*Scedosporium prolificans*, also known as* Scedosporium inflatum*, is a fungus widespread in soil, sewage, and manure. This species is highly virulent and is an emerging opportunistic pathogen found in penetrating injuries in immunocompromised patients. Here we report on an immunocompetent patient with bilateral hip* S. prolificans*-associated osteomyelitis and septic arthritis caused by intentional penetrating trauma. The condition was refractory to initial antimicrobial suppression and surgical irrigation and debridement. Successful outcome was achieved after incorporating a bilateral two-stage total-hip-arthroplasty with Voriconazole-loaded cement and spacer.

## 1. Introduction

*Scedosporium prolificans*, also known as* Scedosporium inflatum*, is a fungus ubiquitous in soil, sewage, potted plants, and manure [1]. This species is an emerging opportunistic pathogen found in penetrating injuries in immunocompromised patients [2].* S. prolificans* infections are resistant to most currently available antifungals thus making treatment options challenging [3]. It was first described in 1984 after isolation from a bone biopsy specimen in an area of osteomyelitis [4]. Treatment of* S. prolificans* infections is complicated by its resistance to most currently available antifungals [3]. Disseminated infections occur more commonly in immunocompromised individuals [5] while localized infections presenting as septic arthritis and osteomyelitis are more common in immunocompetent patients.

## 2. Case Presentation

A 47-year-old male with background history of bilateral hip osteoarthritis presented to the Emergency Department in February 2016 with a one-month history of progressive bilateral groin pain and four-day history of inability to bear weight on the left side. The pain was worse on the left and radiated towards the knees bilaterally. The patient was otherwise well and denied a history of fevers or other constitutional symptoms. Prior to presentation, he was privately managed for five months with multiple intra-articular hip HCLA injections by a radiologist. This provided good effect for four months until one month prior to presentation.

On examination, the patient had hyperaesthesia on palpation over the greater trochanter and groin region. Passive and active movement of both hips were painful with restricted ranges of motion in all directions bilaterally. Neurovascular status was intact. Initial laboratory results revealed a raised CRP (105) with unremarkable FBC, U&E, and LFTs. Neurovascular status was intact. Initial laboratory results revealed a raised CRP (105) with unremarkable FBC, U&E, and LFTs. 24-hour blood cultures,* Chlamydia trachomatis*, gonorrhoea, rheumatoid factor, and anticyclic citrullinated peptide in consideration of rheumatic and infective causes were negative.

**Figure 1:**
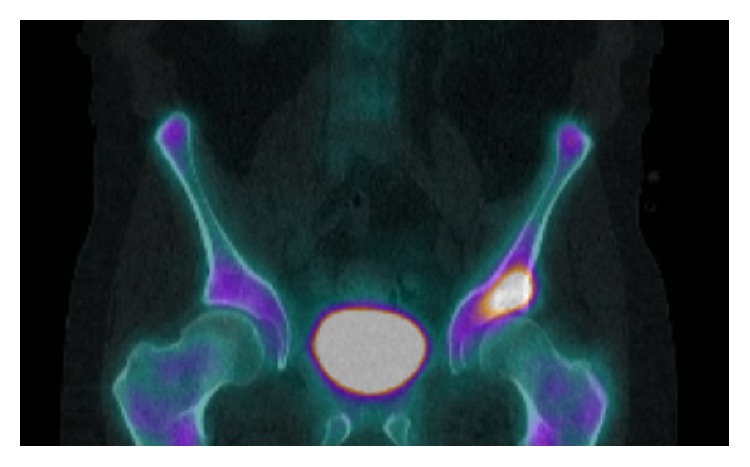
Delayed-phase SPECT/CT displaying increased activity in the left AIIS extending to the superior acetabular rim.

**Figure 2:**
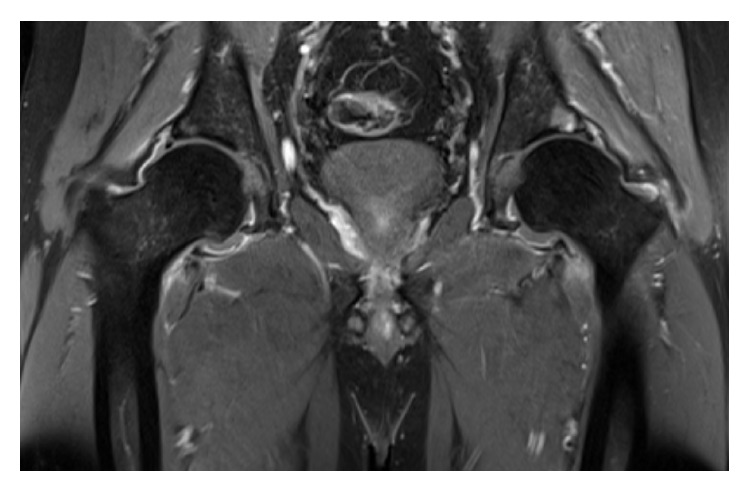
T2 weighted MRI demonstrating a hyperintense focus in the left superior acetabular rim and hip effusion and capsular edema consistent with osteomyelitis and septic arthritis.

Ultrasound guided aspiration of the left hip revealed haemoserous fluid and scant leukocytes but was negative for crystals and bacteria. Interim bone scan (Figure 1) and MRI (Figure 2) showed a probable focus of osteomyelitis within the left anterior inferior iliac spine (AIIS) and small bilateral effusions concerning for superimposed septic arthritis. Bone scintigraphy confirmed activity at the left AIIS (Figure 2). Despite being commenced on IV flucloxacillin, ciprofloxacin, and vancomycin, a low-grade fever developed over the following days. A left hip washout revealed cloudy fluid and exudate with reactive appearance of labrum and capsule. Cultures returned a positive result for* S. prolificans* after five days necessitating the commencement of Voriconazole and Terbinafine.

Following initial improvements, the patient developed worsening left groin pain and rising CRP. A second washout and MRI suggested ongoing left septic arthritis and associated osteomyelitis with contralateral concerning pathology in the right hip. A subsequent bilateral hip washout, left head core decompression, and acetabulum debridement were performed on day 28. Repeat MRI demonstrated worsening left septic arthritis and acetabular osteomyelitis despite ongoing surgical debridement, lavage, and medical therapy. All left-sided intraoperative samples returned positive for* S. prolificans* while right-sided specimens remained negative to date.

**Figure 3:**
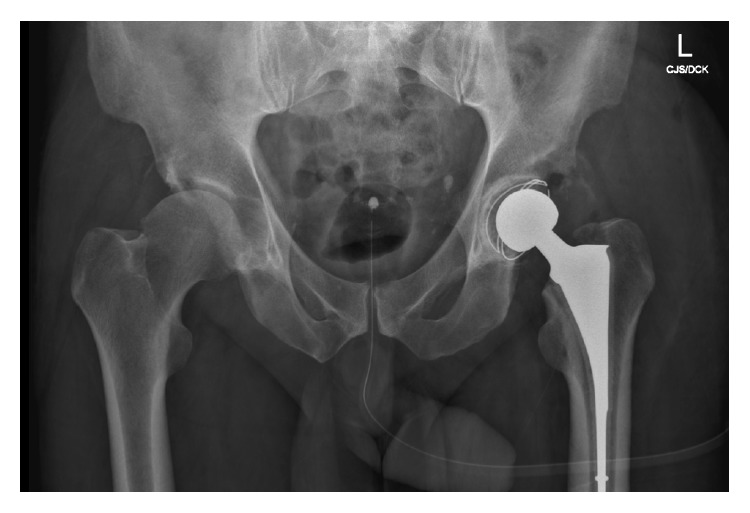
Postoperative radiograph demonstrating first-stage cemented total hip replacement with 48/32 mm Stryker RimFit Acetabular cup and size 9 Biomet Simplex cement spacer.

**Figure 4:**
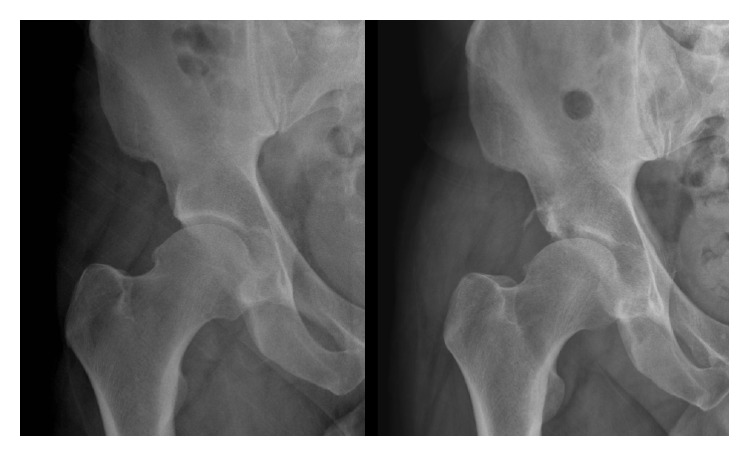
Plain radiograph showing progressive osteolysis of the superior right acetabular rim consistent with chronic infection.

**Figure 5:**
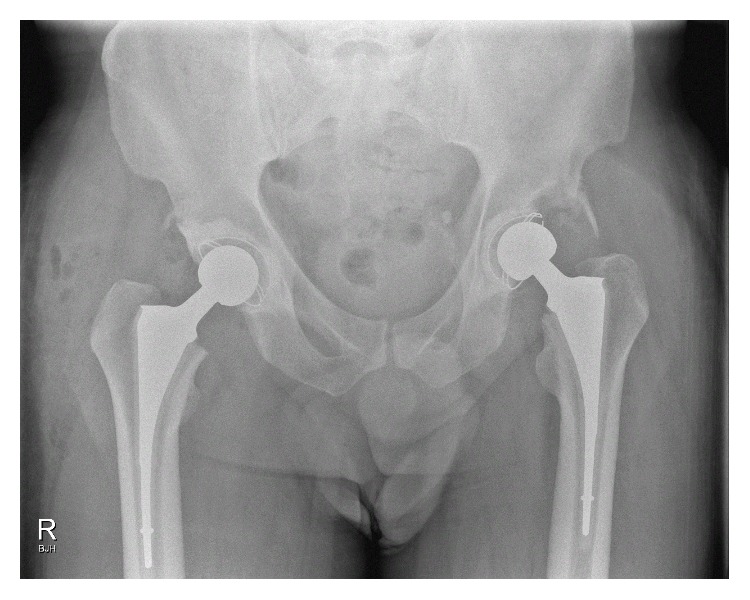
Postoperative radiograph demonstrating bilateral first-stage cemented total hip replacement.

Departmental decision was made to perform a two-stage left total-hip-arthroplasty. The first stage initially involved aggressive acetabular debridement, lavage and reaming. An acetabular cup loaded with 200 mg Voriconazole in Palacos cement and similarly loaded cement spacer were implanted (Figure 3). Prior to home discharge with regular Voriconazole, the patient was mobilising with pain score of 0 on the left and minimal pain on the right side. However, due to interval radiological changes (Figure 4) and right-sided pain at follow-up, a first-stage right hip arthroplasty was performed (Figure 5). The patient is currently seven months after left and 6 months after right first-stage total-hip-arthroplasty. Progression to second-stage total-hip-arthroplasty will be considered following a disease-free period of at least twelve months.

## 3. Discussion

*Scedosporium prolificans*, also known as* Scedosporium inflatum,* is an emerging opportunistic pathogen found in penetrating injuries in immunocompromised patients [2]. Treatment of* S. prolificans* infections is complicated by its resistance to most currently available antifungals [3]. Disseminated infections occur more commonly in immunocompromised individuals [5] while localized infections presenting as septic arthritis and osteomyelitis are more common in immunocompetent patients. We believe the repeated HCLA injections provided a point of entry and a locally immune-deficient environment for the infection to take hold. Corticosteroids may induce an immunosuppressed environment as they inhibit the accumulation of inflammatory cells, phagocytosis, and production of neutrophils and prevent the synthesis and secretion of inflammatory mediators [6, 7]. Previous literature has suggested that the local immunosuppressive effects associated with invasive steroid treatments such as HCLA injection may influence and increase the susceptibility to infection [8]. Thus, any factors which could potentially inhibit the ability of joint to withstand infection should be minimized [8]. This is also in light of the possibility of infection being introduced at the time of invasive therapy. Several case reports of septic arthritis and/or osteomyelitis have appeared in published literature. Here we report on an immunocompetent patient with bilateral hip* S. prolificans-*associated osteomyelitis and septic arthritis treated with a two-stage total-hip-arthroplasty incorporating Voriconazole-loaded cement and spacer.

Although treatment is difficult, localized infections have previously showed response to antifungal therapy and surgical debridement. A recent systematic review included 23 reported cases of* S. prolificans*-associated osteoarticular infections in both immunocompetent or immunocompromised patients [9]. Our review of the English literature revealed 14 case reports of* S. prolificans* infection of the joints (Table 1) in only immunocompetent patients. Reports have seen success with older antifungal agents including Amphotericin B, Ketoconazole, Miconazole, nystatin, 5-fluorocytosine, and fluconazole [3, 10, 11]. Three previous reports have demonstrated satisfactory results with newer antifungal agents Terbinafine/Voriconazole in immunocompetent patients with septic arthritis [12–15]. Another report has shown success in an 8-year-old immunocompetent patient using Hexadecylphosphocholine with Voriconazole/Terbinafine [16]. In vitro synergistic effects have been reported with Voriconazole and Terbinafine, reducing the minimum inhibitory concentration for* S. prolificans *[17, 18]. However, there remains no consensus for the duration of treatment with the Voriconazole/Terbinafine combination for septic arthritis and osteomyelitis caused by* S. prolificans*. Regardless of that, irrigation and surgical debridement are vital components in the eradication of infections in all cases. In cases unresponsive to antifungal treatment and surgical debridement, arthrodesis [15] alongside radical excisions and amputations has been necessitated [19].

This is the first described case to our knowledge of* S. prolificans*-associated septic arthritis and osteomyelitis treated using a two-stage hip arthroplasty with Voriconazole-loaded cement and spacer. Studies have confirmed that Voriconazole is stable at high temperatures and therefore suitable to be used with Palacos cement [20]. Previous studies have suggested that the elution of Voriconazole from bone cement would be able to maintain a minimum inhibitory concentration for* S. prolificans* [21]. Higher antimicrobial content has been shown to increase cement porosity and thus elution. However, concern exists as load-bearing strength potential of cement and hardening time is consequently reduced. This affects the time required for spacer preparation. Therefore, a balance between these factors needs to be considered.

The objective in this case, to permanently eradicate the infection and restore function to the patient, was successful. This case therefore highlights the need to consider alternative avenues before undertaking more drastic measures such as arthrodesis or amputation for* S. prolificans* septic arthritis and osteomyelitis.

## Figures and Tables

**Table 1:** Reported *Scedosporium prolificans*-associated osteomyelitis and/or septic arthritis infections from penetrating trauma in immunocompetent patients.

Age/gender	Location	Mechanism of introduction	Site/presentation	Treatments used	Final Outcome	Author/Year
6/M	North America	Penetrating injury	Foot osteomyelitis	Surgical debridement, Amphotericin B, Ketoconazole, Miconazole,	Improvement	Taj-Aldeen et al. 2015 [9]

3/M	South America	Trauma	Knee septic arthritis	Surgery, Ketoconazole, Amphotericin B, Ketoconazole, intra-articular Amphotericin B, intra-articular Miconazole	Amputation	Wilson et al. 1990 [19]

5/M	North America	Penetrating trauma from thorn	Knee septic arthritis	Surgery, Amphotericin B 5-FC, intra-articular Amphotericin B, Miconazole, Ketoconazole	Improvement	Wilson et al. 1990 [19]

54/M	North America	Trauma from axe	Knee arthritis	Surgery, Amphotericin B, Ketoconazole; Miconazole	Improvement	Wilson et al. 1990 [19]

6/M	North America	Penetrating injury from nail	Foot osteomyelitis	Surgical debridement	Improvement	Wilson et al. 1990 [19]

6/M	North America	Penetrating injury from nail	Foot osteomyelitis	Surgery, Amphotericin B, Ketoconazole	Improvement	Wilson et al. 1990 [19]

35/M	North America	Penetrating injury/IV drug use	Hip septic arthritis	Joint drainage, Amphotericin B, 5-fluorocytosine	Improvement	Wilson et al. 1990 [19]

11/M	Australia	Laceration	Ankle septic arthritis	Surgical debridement, Amphotericin B, Itraconazole	Improvement	Wood et al. 1992 [11]

5/M	North America	Penetrating injury from nail	Foot osteomyelitis	Surgical debridement, polyhexamethylene, biguanide, Voriconazole, caspofungin	Improvement	Steinbach et al. 2003 [3]

9/M	Sweden	Penetrating injury from thorn	Knee osteomyelitis	Surgical debridement, cefuroxime, Amphotericin B, Itraconazole, Voriconazole	Improvement with arthrodesis	Studahl et al. 2003 [15]

5/M	Australia	Ankle abrasion due to trauma	Ankle septic arthritis	Surgical debridement, Amphotericin B, Itraconazole, Terbinafine, Voriconazole	Improvement	Dalton et al. 2006 [13]

8/F	Australia	Trauma from tractor	Hip osteomyelitis and hip septic arthritis	Surgical debridement, Hexadecylphosphocholine, Terbinafine, Voriconazole	Improvement	Kesson et al. 2009 [16]

4/M	United Kingdom	Penetrating injury from thorn	Foot osteomyelitis	Surgical debridement, Voriconazole, Terbinafine	Improvement	Bhagavatula et al. 2014 [14]

54/M	Australia	Penetrating injury from HCLA injections	Hip osteomyelitis and hip septic arthritis	2-stage total-hip-arthroplasty with Voriconazole-loaded cement and spacer, Voriconazole, Terbinafine	Improvement	Daniele et al. 2016
